# Robot-assisted partial nephrectomy for TFE3-rearranged renal cell carcinoma: a case report and literature review

**DOI:** 10.3389/fonc.2026.1740901

**Published:** 2026-03-20

**Authors:** Biao Jiang, Shihang Zhang, Hailin Luo, Chuance Du

**Affiliations:** Department of Urology, Ganzhou People’s Hospital, Ganzhou, Jiangxi, China

**Keywords:** partial nephrectomy, prognosis, renal cell carcinoma, TFE3, Xp11.2 translocation

## Abstract

**Background:**

TFE3-rearranged renal cell carcinoma (TFE3-RCC) is a rare RCC subtype driven by translocation involving Xp11.2 and TFE3 gene fusion. The management of TFE3-RCC is similar to that of other RCC subtypes and primarily involves surgical resection, targeted therapy, and immunotherapy, with surgical excision serving as the mainstay of treatment for localized disease. This study presents a rare case and demonstrates the feasibility and efficacy of robot-assisted partial nephrectomy (RAPN) in the treatment of localized TFE3-RCC, indicating that with meticulous planning and selection, RAPN could achieve a favorable prognosis in cases of localized TFE3-RCC.

**Case presentation:**

We report a case of TFE3 gene fusion-associated renal cell carcinoma (RCC) in a 28-year-old woman who presented with a history of recurrent left flank pain for over two years, which had intensified over the preceding month. Two years earlier, in July 2022, the patient had been diagnosed with a “left renal cyst” at a local institution and received symptomatic treatment. In August 2024, due to the recent aggravation of symptoms, she was admitted for further evaluation. Abdominal contrast-enhanced computed tomography (CT) revealed a Bosniak category III cystic renal lesion, raising suspicion for renal cell carcinoma, measuring approximately 2.1 × 2.4 × 2.1 cm. In September 2024, the patient underwent robot-assisted laparoscopic partial nephrectomy for tumor excision. Histopathological analysis was suggestive of RCC with Xp11.2 translocation/TFE3 gene fusion. The pathological stage was classified as pT1aN0M0, and surgical margins were negative for malignancy. At the one-year follow-up, the patient recovered well without receiving any adjuvant therapy.

**Conclusion:**

This study elucidates the natural history, diagnosis, and management of TFE3-RCC, underscoring the critical importance of early surgical intervention. TFE3-RCC demonstrates marked aggressiveness, characterized by high recurrence rates and a poor prognosis in adult patients. Surgical resection serves as the mainstay of treatment for localized disease. Given its aggressive nature and substantial risk of recurrence and metastasis, diligent long-term follow-up is essential. For advanced or metastatic disease, targeted therapy and immunotherapy emerge as potential treatment options.

## 1 Introduction

TFE3-rearranged renal cell carcinoma (TFE3-RCC) is an exceptionally rare subtype of renal cell carcinoma (RCC), initially delineated by the World Health Organization (WHO) in 2004 (1). Under the 2016 WHO classification of renal tumors, TFE3-RCC was categorized as part of the MiT family translocation renal cell carcinomas (2). The MiT family encompasses four genes—TFE3, TFEB, TFEC, and MITF—that are pivotal in regulating diverse cellular processes, including cell differentiation, autophagy, and lysosome biogenesis (3, 4). A notable progression was achieved with the 2022 WHO renal tumor classification update, which incorporated molecular markers into the traditional morphological framework, encompassing entities such as TFE3-rearranged RCC, TFEB-altered RCC, ALK-rearranged RCC, and ELOC-mutated RCC (5). TFE3-rearranged RCC is now considered the most common subtype within this category.

TFE3-rearranged RCC demonstrates a relatively low incidence, accounting for approximately 1-4% of adult RCC cases, but represents a substantially higher proportion (20-75%) in pediatric RCC cohorts (6, 7). The meta-analysis by Cheng et al. (7) indicated a significantly higher incidence in female patients compared to males, which may be associated with the characteristic Xp11.2 chromosomal translocation, and this genetic rearrangement might lead to the sex-based disparity. However, some studies have indicated that the sex difference in TFE3-rearranged RCC is not statistically significant (8).

TFE3-rearranged renal cell carcinoma (RCC) is highly aggressive and often exhibits resistance to conventional chemotherapy and radiotherapy, which ultimately leads to a poor prognosis (7). Consequently, accurate diagnosis and optimal surgical management are critically important. For localized RCC, partial nephrectomy (PN) has become the preferred treatment for clinical T1 (cT1) renal tumors (9). Compared to radical nephrectomy (RN), PN not only preserves renal function but also ensures effective oncological outcomes (10, 11). With the advancement and application of robot-assisted techniques, the indications for PN have been further expanded, particularly in managing more complex cases and larger renal masses (12–14). Equipped with an array of integrated technical tools, the robotic console significantly improves surgical accuracy and aids in formulating personalized treatment regimens. In contrast to open surgery, robot-assisted laparoscopic partial nephrectomy (RAPN) is widely utilized in adults with localized RCC due to its favorable perioperative outcomes and superior renal function preservation (15). For patients with advanced or metastatic TFE3-rearranged RCC, targeted therapy and immunotherapy represent key therapeutic options.

This study presents a rare case of TFE3-rearranged renal cell carcinoma (RCC). The patient was found to have an Xp11.2 translocation resulting in TFE3 gene fusion over the course of approximately two years. After excluding surgical contraindications, the patient successfully underwent robot-assisted laparoscopic partial nephrectomy. Postoperative surveillance at the one-year follow-up revealed no evidence of tumor recurrence. By reviewing contemporary progress in the diagnosis and management of TFE3-RCC, this article aims to offer a valuable reference to guide future investigations into this distinct RCC subtype.

## 2 Case presentation

**Figure 1:**
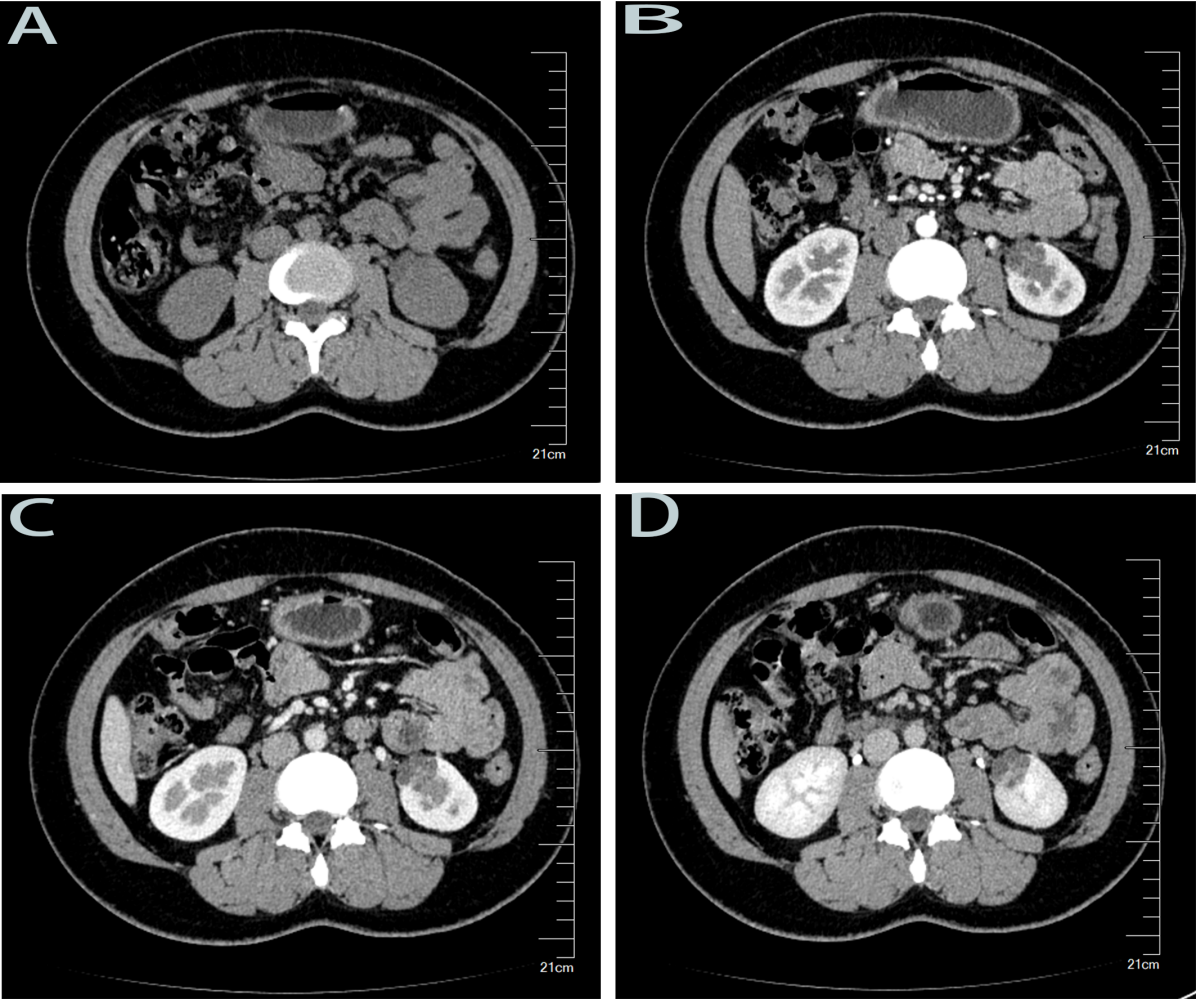
The CT imaging findings (non-contrast and contrast-enhanced scans of the whole abdomen) for this case upon admission are as follows: **(A)** Non-contrast CT scan: A cystic, slightly hypodense shadow is observed in the left kidney. **(B)** Contrast-enhanced CT scan, arterial phase: Mild to moderate enhancement of the cystic wall and septa within the mass is visible. **(C)** Contrast-enhanced CT scan, venous phase: The mass appears lobulated and contains multiple internal septations. **(D)** Contrast-enhanced CT scan, excretory phase: Patchy high-density shadows are noted in the left renal sinus.

A 26-year-old female patient presented to the urology outpatient department of a local hospital in July 2022 with lower back pain. Abdominal CT suggested the presence of a left renal cyst. The patient received symptomatic treatment, which relieved her symptoms. In August 2024, two years later, she returned to our hospital’s urology outpatient clinic due to aggravated lower back pain. The patient reported that the pain had no obvious cause and was worsening progressively. Her medical and family histories were unremarkable, with no familial cancers or hereditary diseases. Routine laboratory tests, including complete blood count, urinalysis, β-HCG, and assessments of renal, hepatic, and adrenal function, showed no abnormalities. Pregnancy was excluded. The results indicated a urinary tract infection, while other laboratory parameters were within normal limits. Non-contrast and contrast-enhanced computed tomography of the whole abdomen identified a lesion with cystic changes at the lower pole of the left kidney. This lesion was classified as a Bosniak category III renal cyst and was considered suspicious for renal cell carcinoma (**Figure 1**). Other imaging examinations, including X-ray and MRI, revealed no additional abnormalities. Two weeks later, after the urinary tract infection was controlled, the patient was scheduled for surgical intervention. Preoperative evaluation identified no contraindications to surgery.

To ensure the successful execution of the surgery, a multidisciplinary team discussion was held. Based on preoperative imaging, which showed no evidence of distant metastasis, and considering the patient’s R.E.N.A.L. score of 3, the experts unanimously agreed to proceed with a robot-assisted laparoscopic partial nephrectomy. The patient provided informed consent, and the surgery was scheduled for September 6, 2024.During the procedure, the renal fascia was dissected, the left renal artery was identified, and adhesions were carefully released. A 3 cm cystic mass was excised from the left kidney after mobilizing the surrounding structures. The renal artery was clamped, and the warm ischemia time was 18 minutes. Blood loss was minimal, approximately 45mL, with no need for transfusion. The entire procedure lasted 97 minutes, and the surgery was successfully completed under general anesthesia.

**Figure 2:**
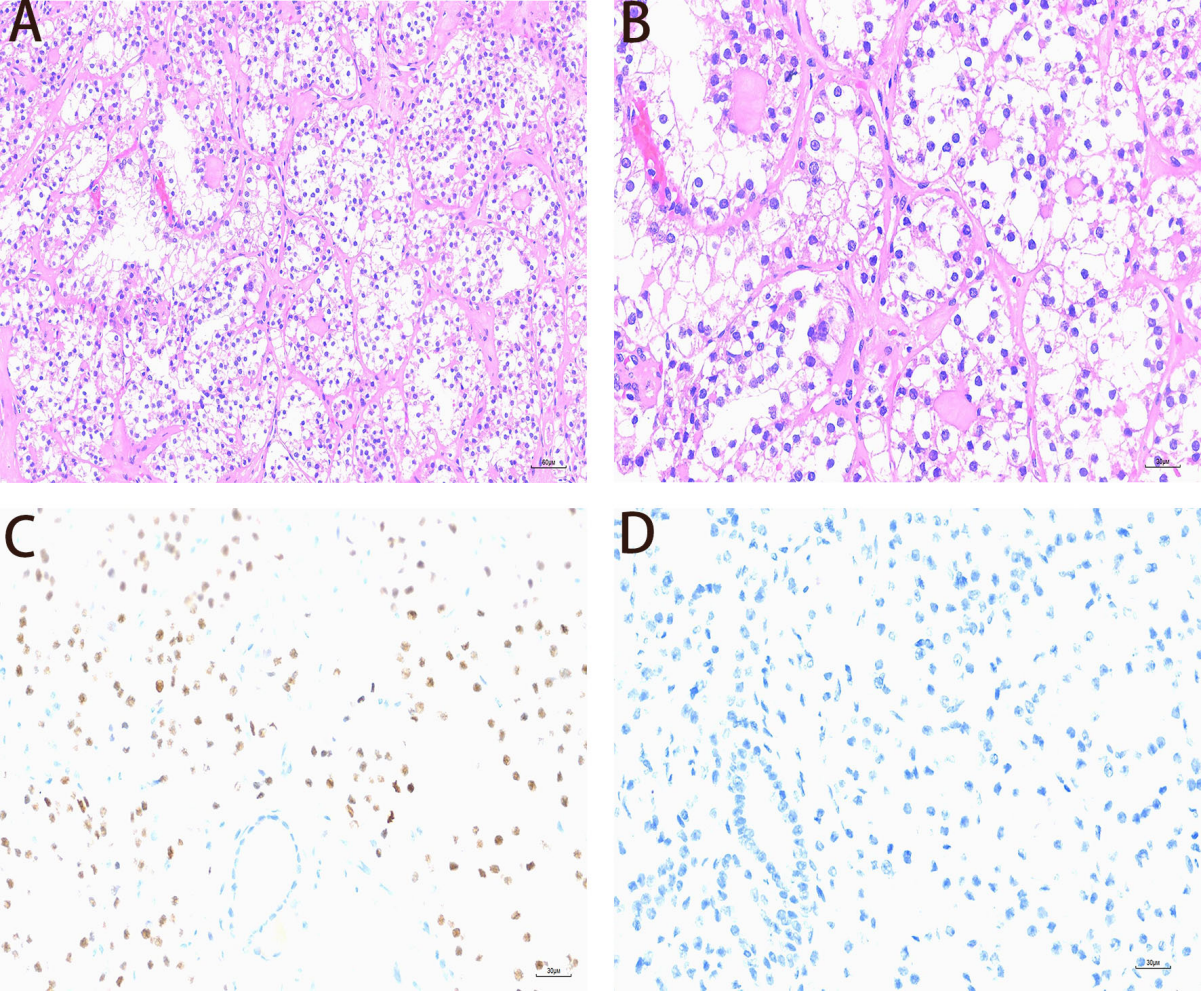
Pathological and Immunohistochemical Findings of the Case **(A)** The tumor cells are arranged in tubular, papillary, and nested patterns, accompanied by abundant sinusoidal vessels (Hematoxylin and Eosin [H&E], ×20). **(B)** The tumor cells exhibit significant atypia, with clear or eosinophilic cytoplasm and prominent nucleoli (H&E, ×40). **(C)** Expression of the transcription factor binding to IGHM enhancer 3 (TFE3) in the tumor tissue (IHC, ×40). **(D)** Absence of HMB-45 expression in the tumor tissue (IHC, ×40).

The resected specimen was a greyish-white and greyish-red mass measuring 2.5 × 2.5 × 1.8 cm. Postoperative pathological examination revealed a multicystic tumor. The inner cystic walls exhibited small nests or papillary growths of tumor cells. These cells featured roundish nuclei, prominent nucleoli, and clear cytoplasm, Psammoma bodies were observed within the stroma (**Figures 2A, B**). Immunohistochemical staining yielded the following results(**Figures 2C, D**): Ki67 (approximately 2%+), p504S (+), Pax-8 (+), CD10 (–), CD117 (+), CK7 (–), CK (partial +), Vimentin (+), CA-IX (–), GATA3 (–), CK34βE12 (–), ALK (5A4) (focal +), SDHB (+), TFE3 (+), Melan-A (+), HMB45 (–), FH (+). These findings support the diagnosis of TFE3-rearranged renal cell carcinoma. The patient recovered well postoperatively and was discharged on the 10th postoperative day. Neither radiotherapy nor chemotherapy was administered.

**Figure 3:**
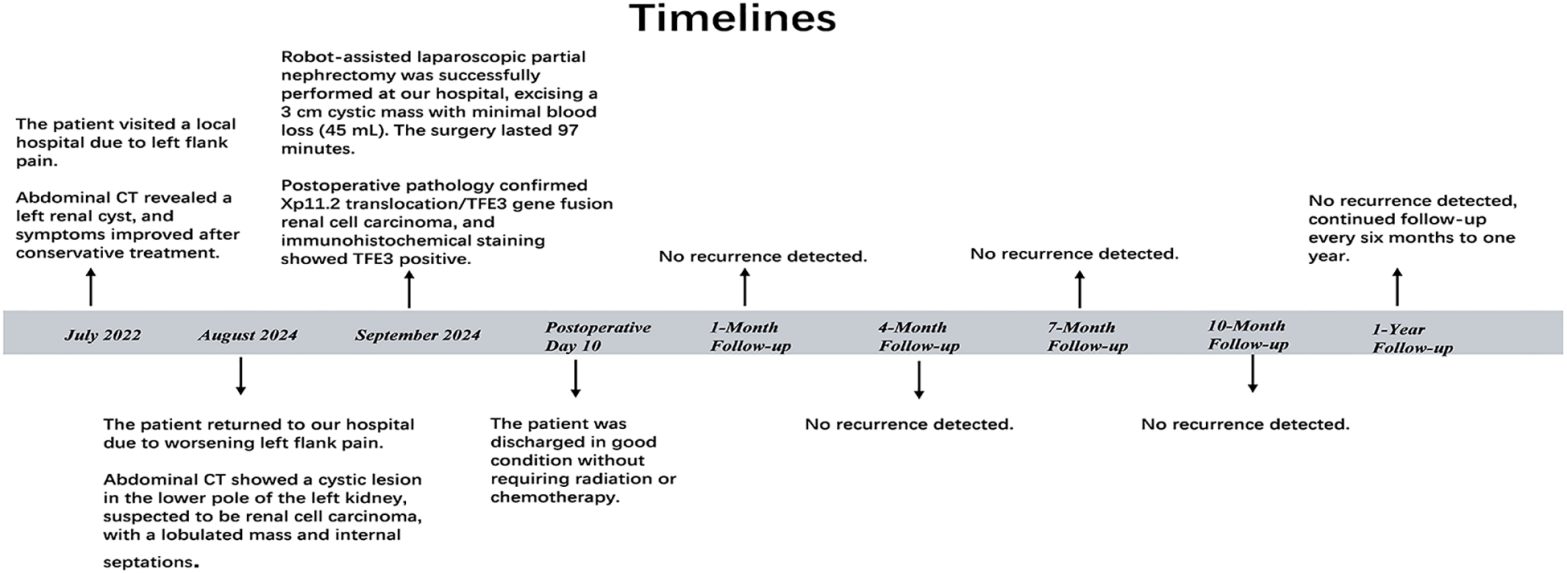
Timelines.

**Figure 4:**
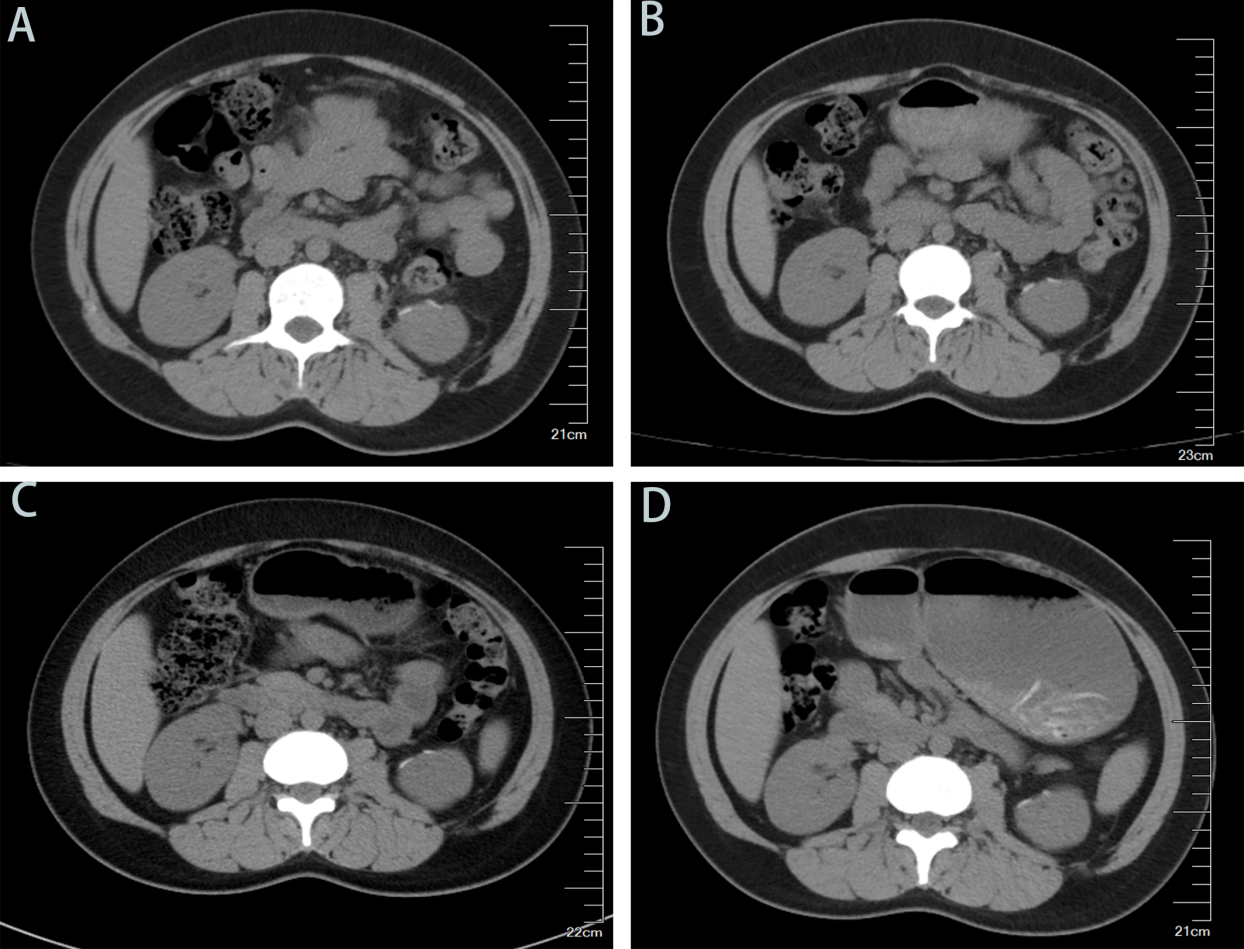
Follow-up Computed Tomography (Non-contrast CT of the Whole Abdomen) Findings. **(A)** 1-month postoperative non-contrast abdominal CT scan: Showing no evidence of tumor recurrence or metastasis. **(B)** 4-month postoperative non-contrast abdominal CT scan: Showing no evidence of tumor recurrence or metastasis. **(C)**7-month postoperative non-contrast abdominal CT scan: Showing no evidence of tumor recurrence or metastasis. **(D)** 10-month postoperative non-contrast abdominal CT scan: Showing no evidence of tumor recurrence or metastasis.

The first follow-up took place one month after surgery, followed by subsequent visits every three months. After the first year, follow-up visits will be conducted every six months to one year (**Figure 3**). Beginning at five years post-surgery, follow-up visits will be scheduled every two years. More than one year after surgery, the patient remains free of tumor recurrence during routine follow-up visits (**Figure 4**). The workup, including physical examination, laboratory tests, and non-contrast CT scans of the chest and whole abdomen, showed no abnormalities. Continued long-term follow-up is advised to monitor the patient’s medium- and long-term prognosis.

## 3 Discussion

Similar to other forms of RCC, most patients with TFE3-RCC do not present with the renal cancer triad. Approximately one-third of patients are asymptomatic and do not exhibit significant pain, showing only a renal mass. Some patients may develop systemic symptoms such as weight loss, fever, or fatigue due to tumor metastasis. Our case aligns with these findings: the patient had no macroscopic hematuria, no palpable abdominal mass, and presented solely with recurrent flank pain. Due to the lack of specific symptoms, many patients are not diagnosed early, and the tumor is often incidentally detected during imaging studies. Radiologically, the tumor is commonly described as a hypodense or multiloculated cystic lesion, with calcifications observed in some cases. Contrast-enhanced CT in our patient showed a lobulated mass with internal septations, which is consistent with the typical imaging features of TFE3-RCC.

TFE3-RCC exhibits high invasiveness, with approximately 47% of patients developing metastasis within 24 months post-surgery (16). A retrospective analysis demonstrated that patients with TFE3-rearranged RCC had a median overall survival of 74.5 months, with higher T stage and older age at diagnosis significantly associated with prognosis (17). Pathological diagnosis of TFE3-RCC requires integration of histological features, immunohistochemical markers, and molecular testing results. Microscopically, tumor cells often exhibit papillary, acinar, or nests of cells growth patterns with eosinophilic to clear cytoplasm, and prominent nucleoli are visible in some areas. TFE3-rearranged renal cell carcinoma (RCC) typically presents with a distinctive set of immunohistochemical markers. Commonly observed positive markers include PAX8, Vimentin, P504S, TFE3 protein, and CD10. In contrast, cytokeratin 7 (CK7) and carbonic anhydrase IX (CA IX) are generally negative (18). In this case, the patient exhibited positive immunostaining for p504S, Pax-8, Vimentin, TFE3 protein, and Mel-A. FISH and PCR analysis are reliable methods for confirming TFE3 gene rearrangement (19, 20). In the differential diagnosis, TFE3-RCC must be distinguished from other renal cell carcinoma subtypes with similar morphological features, such as clear cell RCC and papillary RCC. Clear cell renal cell carcinoma typically exhibits clear cytoplasm and positive immunohistochemical staining for CAIX, whereas TFE3-positive renal cell carcinoma is CAIX-negative. Additionally, HMB45 (–) helps to exclude TFE3-rearranged PEComa tumors (21).Therefore, integrating the findings from immunohistochemistry (IHC) and molecular testing is essential for the diagnosis of TFE3-RCC (22).

The management of TFE3-rearranged RCC (TFE3-RCC) is similar to that of other RCC subtypes, primarily involving surgical resection, targeted therapy, and immunotherapy. The European Association of Urology guidelines for RCC state that, for localized TFE3-rearranged RCC, surgery remains the sole curative treatment (9). Currently, the prognosis for early-stage, localized TFE3-RCC is relatively favorable, with treatment typically centered on radical nephrectomy or partial nephrectomy. While traditional open surgery is applicable to most renal tumors, robot-assisted procedures are increasingly utilized with advances in minimally invasive techniques. Robotic technology offers advantages such as magnified visibility, optimized ergonomics, and the use of Endo Wrist instruments, enabling surgeons to perform tissue dissection and tumor resection with enhanced precision. This approach has been shown to significantly reduce perioperative complications, including blood loss, hospital stay, and overall complication rates (23, 24). Following a preoperative multidisciplinary discussion, the consensus among experts was that, based on the imaging and laboratory findings along with the patient’s specific condition, robot-assisted laparoscopic partial nephrectomy represented the most effective treatment strategy. Postoperatively, the patient recovered well with no signs of recurrence or metastasis.

This case highlights the feasibility and effectiveness of robotic-assisted partial nephrectomy (RAPN) for treating TFE3-rearranged RCC, a rare and less-studied subtype of renal cell carcinoma. While existing guidelines primarily advocate for surgical resection in localized RCC, the use of RAPN in this case demonstrates its potential advantages, including minimal invasiveness and quicker recovery. This case supports the growing body of evidence favoring RAPN for complex renal tumors and contributes to the ongoing discussion regarding its application in rare RCC subtypes, providing a foundation for future studies in this area.

For patients with advanced TFE3-rearranged RCC (TFE3-RCC), targeted therapy and immunotherapy serve as the primary treatment strategies. Cytoreductive surgery combined with targeted or immunotherapy has also been employed in the management of advanced cases (25, 26). Although immune checkpoint inhibitors (ICIs) and tyrosine kinase inhibitors (TKIs) generally demonstrate poor responses in most TFE3-RCC patients, studies suggest that certain subtypes (e.g., those with ASPL-TFE3 fusion) may exhibit a better response to combination therapy with ICIs and TKIs (27–31). Targeted therapy and immunotherapy provide new treatment options for TFE3-RCC patients. However, drug responses exhibit substantial variation among individuals, necessitating the development of personalized treatment plans based on specific patient profiles, combined with lifelong follow-up to assess long-term prognosis.

TFE3-rearranged RCC (TFE3-RCC) is clinically rare, and the factors influencing its prognosis have remained controversial. Potential prognostic factors may include age, AJCC stage, tumor size, lymph node infiltration, neutrophil-to-lymphocyte ratio (NLR), and platelet-to-lymphocyte ratio (PLR). Recent studies suggest that lymph node infiltration, tyrosine kinase inhibitor therapy, and surgical approach are independent risk factors for postoperative progression-free survival (PFS), while factors like tumor stage, age, hemorrhage, and necrosis show no significant association with PFS (32–34). The discrepancies in findings across different studies may be attributed to variations in sample size, clinical characteristics, and research methodologies. Consequently, the prognostic factors for TFE3-RCC require further validation through prospective, large-sample studies.

This study has several limitations. First, as a case report involving only a single patient, the generalizability and broader applicability of the findings are limited and may not represent the clinical characteristics and treatment outcomes of all patients with TFE3-rearranged renal cell carcinoma. Second, although the patient showed no evidence of recurrence or metastasis during the one-year postoperative follow-up, the lack of long-term follow-up data precludes assessment of long-term prognosis. Third, the diagnosis is primarily based on the typical clinical presentation, auxiliary examinations, and immunohistochemical results. Due to the patient’s personal financial constraints, they declined further confirmatory tests (such as FISH) at their own expense; therefore, direct molecular evidence of gene rearrangement could not be obtained. Nevertheless, the existing evidence is suggestive of this diagnosis. Furthermore, the study did not evaluate the comparative efficacy of other treatment modalities, such as targeted therapy and immunotherapy. Accordingly, future multi-center, large-sample studies are necessary to validate treatment outcomes and further refine the management strategy for TFE3-rearranged renal cell carcinoma (TFE3-RCC).

## 4 Patient perspective

I was initially concerned about my worsening left flank pain and, after discussing my options, I chose robot-assisted laparoscopic partial nephrectomy (RAPN) for treatment. The surgery went well, and I’m thankful for the absence of major complications. My recovery was relatively quick, allowing me to return to normal activities sooner than expected. During follow-up visits, I was anxious about recurrence, but each check-up reassured me as no signs of recurrence were found. I feel supported by my medical team and remain committed to my follow-up appointments.

## 5 Conclusion

TFE3-rearranged RCC (TFE3-RCC) is a rare and aggressive subtype of renal cell carcinoma, typically associated with a poor prognosis. For localized, early-stage TFE3-RCC, robot-assisted laparoscopic partial nephrectomy serves as an effective therapeutic approach. In advanced TFE3-RCC, targeted therapy and immunotherapy provide new treatment options. However, due to significant interpatient variability, treatment plans should be individualized based on clinical circumstances and supplemented with lifelong follow-up to assess long-term outcomes.

## Data Availability

The original contributions presented in the study are included in the article/supplementary material. Further inquiries can be directed to the corresponding author.
